# Corticomotor reorganization during short‐term visuomotor training in the lower back: A randomized controlled study

**DOI:** 10.1002/brb3.1702

**Published:** 2020-07-07

**Authors:** Rocco Cavaleri, Lucy S. Chipchase, Hugo Massé‐Alarie, Siobhan M. Schabrun, Muath A. Shraim, Paul W. Hodges

**Affiliations:** ^1^ School of Health Sciences Western Sydney University Campbelltown New South Wales Australia; ^2^ College of Nursing and Health Sciences Flinders University Adelaide South Australia Australia; ^3^ CIRRIS Research Centre Department of Rehabilitation Laval University Quebec Canada; ^4^ Faculty of Health and Behavioural Sciences The University of Queensland Brisbane Queensland Australia; ^5^ Neuroscience Research Australia Randwick New South Wales Australia

**Keywords:** corticomotor reorganization, lower back, motor learning, TMS

## Abstract

**Introduction:**

Accumulating evidence suggests that motor skill training is associated with structural and functional reorganization of the primary motor cortex. However, previous studies have focussed primarily upon the upper limb, and it is unclear whether comparable reorganization occurs following training of other regions, such as the lower back. Although this holds important implications for rehabilitation, no studies have examined corticomotor adaptations following short‐term motor training in the lower back.

**Method:**

The aims of this study were to (a) determine whether a short‐term lumbopelvic tilt visuomotor task induced reorganization of the corticomotor representations of lower back muscles, (b) quantify the variability of corticomotor responses to motor training, and (c) determine whether any improvements in task performance were correlated with corticomotor reorganization. Participants were allocated randomly to perform a lumbopelvic tilt motor training task (*n* = 15) or a finger abduction control task involving no lumbopelvic movement (*n* = 15). Transcranial magnetic stimulation was used to map corticomotor representations of the lumbar erector spinae before, during, and after repeated performance of the allocated task.

**Results:**

No relationship between corticomotor reorganization and improved task performance was identified. Substantial variability was observed in terms of corticomotor responses to motor training, with approximately 50% of participants showing no corticomotor reorganization despite significant improvements in task performance.

**Conclusion:**

These findings suggest that short‐term improvements in lower back visuomotor task performance may be driven by changes in remote subcortical and/or spinal networks rather than adaptations in corticomotor pathways. However, further research using tasks of varying complexities and durations is required to confirm this hypothesis.

## Graphical abstract

Accumulating evidence suggests that motor skill training is associated with structural and functional reorganization of the primary motor cortex. However, previous studies have focussed primarily upon the upper limb, and it is unclear whether comparable reorganization occurs following training of other regions, such as the lower back. The aims of this study were therefore to (a) determine whether a lumbopelvic tilt motor training task induced reorganization of the corticomotor representations of lower back muscles, (b) quantify the variability of corticomotor responses to motor training, and (c) determine whether any improvements in task performance were correlated with corticomotor reorganization. No relationship between corticomotor reorganization and improvements in task performance was identified, suggesting that short‐term improvements in lower back performance may be driven by changes in remote subcortical and/or spinal networks rather than adaptations in corticomotor pathways.

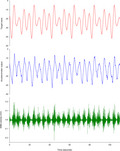

## 1 INTRODUCTION

The acquisition of novel motor skills is associated with structural and functional reorganization of the primary motor cortex (M1) (Adkins, Boychuk, Remple, & Kleim, 2006; Ljubisavljevic, 2006; Ruffino, Papaxanthis, & Lebon, 2017). In humans, transcranial magnetic stimulation (TMS) has been used to demonstrate corticomotor reorganization following a variety of motor skill training tasks. For example, individuals trained to perform piano exercises show greater excitability of M1 digit representations for the trained hand compared to untrained controls (Pascual‐Leone et al., 1995). This is consistent with experimental studies that demonstrate M1 reorganization in response to upper limb motor training (Classen, Liepert, Wise, Hallett, & Cohen, 1998; Lotze, Braun, Birbaumer, Anders, & Cohen, 2003; Muellbacher, Ziemann, Boroojerdi, Cohen, & Hallett, 2001; Pascual‐Leone, Grafman, & Hallett, 1994; Pascual‐Leone et al., 1995). Cross‐sectional studies reveal similar findings, with braille readers and tennis players possessing larger hand representations in M1 than people not proficient in these skills (Pascual, Wassermann, Sadato, & Hallett, 1995; Pascual‐Leone et al., 1993; Pearce, Thickbroom, Byrnes, & Mastaglia, 2000). These observations support animal work demonstrating greater M1 synaptogenesis following reaching and manipulation training than nonskilled repetitive movement (Allred & Jones, 2004; Bury & Jones, 2002; Greenough, Larson, & Withers, 1985; Kleim et al., 2002, 2004).

The degree of M1 reorganization associated with changes in motor performance may depend upon the region being trained. Training‐induced M1 changes have been shown to be graded from distal to proximal, with hand representations showing greater reorganization than upper arm representations (Krutky & Perreault, 2007). The potential for region‐specific differences in M1 reorganization is supported by findings that interventions, such as theta burst stimulation (Martin, Gandevia, & Taylor, 2006) and ipsilateral hand movement (Sohn, Jung, Kaelin‐Lang, & Hallett, 2003), induce more effective corticomotor inhibition in distal than proximal muscle representations. Structural and functional differences across M1 representations may contribute to this region‐dependent reorganization. For example, distal muscles, involved in fine motor control, possess larger and more excitable M1 representations than those for proximal muscles that contribute to limb positioning (Palmer & Ashby, 1992; Wassermann, McShane, Hallett, & Cohen, 1992). These findings suggest that neural contributions to improved motor performance may differ between muscle groups (Krutky & Perreault, 2007). However, as studies of motor training have focussed primarily on the upper limb (Adkins et al., 2006; Carson, Ruddy, & McNickle, 2016; Ljubisavljevic, 2006), it remains unclear whether the potential for corticomotor reorganization following training is limited in other proximal and axial regions, such as the lower back.

Lower back muscles are functionally distinct from those of the limbs, providing a greater contribution to postural functions than fine motor control (Kaigle, Holm, & Hansson, 1995; Tsao, Druitt, Schollum, & Hodges, 2010; Wilke, Wolf, Claes, Arand, & Wiesend, 1995). Trunk muscles have a greater proportion of ipsilateral projections from M1 than upper limb muscles (Strutton et al., 2004), and motor control of these regions involves different pathways in the basal ganglia (Visser et al., 2008). There are also fewer corticospinal projections to the lower back than to the hand, with these projections arising from a smaller cortical area (Cheyne, Kristeva, & Deecke, 1991; Penfield & Boldrey, 1937). Animal studies suggest that lower back muscles receive greater input from subcortical sites when compared to upper limb muscles, which demonstrate cortical dominance (Deliagina, Beloozerova, Zelenin, & Orlovsky, 2008; Galea, Hammar, Nilsson, & Jankowska, 2010; Lemon, Kirkwood, Maier, Nakajima, & Nathan, 2004). These features of back muscle control might underlie differences in the potential role and influence of corticomotor reorganization during motor skill acquisition. Although this holds implications for rehabilitation and motor retraining, no studies have examined corticomotor adaptations following short‐term motor training, or the relationship between these factors, in the lower back.

This study aimed to (a) determine whether short‐term motor training (i.e., a lumbopelvic tilt visuomotor task) induced reorganization of the corticomotor representations of lower back muscles, (b) quantify the degree of variation of corticomotor responses to visuomotor training in the lower back, and (c) determine whether improvements in task performance were correlated with corticomotor reorganization. A rapid TMS mapping technique was employed to probe corticomotor reorganization (Cavaleri, Schabrun, & Chipchase, 2018; van de Ruit, Perenboom, & Grey, 2015). The short duration of this technique allowed investigation of transient changes in the size and distribution of corticomotor representations that might be induced by short‐term training, but not feasibly assessed using conventional methods. Given previous observations regarding the corticomotor reorganization of proximal upper limb muscles following motor training, we hypothesized that lower back representations would exhibit only small changes in excitability or organization following motor training.

## 2 MATERIALS AND METHODS

### 2.1 Participants

Thirty healthy individuals (20 male, 10 female) participated. Participants were excluded if they presented with acute pain, a history of chronic pain, neurological disorders, musculoskeletal impairments, or contraindications to TMS identified using the Transcranial Magnetic Stimulation Adult Safety Screen questionnaire (Rossi, Hallett, Rossini, & Pascual‐Leone, 2009, 2011). Participants provided written informed consent prior to testing. Experimental procedures were approved by the local institutional Human Research Ethics Committees and performed in accordance with the Declaration of Helsinki.

### 2.2 Experimental protocol

**FIGURE 1:**
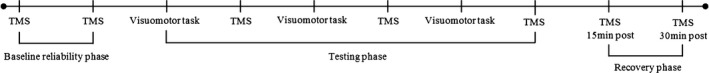
Experimental protocol. TMS, transcranial magnetic stimulation

Participants attended a single experimental session and were requested to refrain from caffeine, alcohol, or exhaustive exercise from 12 hr prior to testing. Before testing, a random number generator was used to randomly allocate participants to either the experimental task (lumbopelvic tilt motor training task) or a control task (repeated finger abduction) involving no lumbopelvic movement. The experimental session was divided into three phases: Baseline, Testing, and Recovery (Figure 1). During the Baseline phase, the corticomotor representation of the lumbar erector spinae (LES) muscle was mapped twice using TMS. This was done to examine the within‐session reliability of map features in the absence of the motor training task. During the Testing phase, participants performed one of the two training tasks for a total of 15 min, which was divided into three 5‐min training blocks. Maps of the LES representation were acquired after each training block to examine potential changes in map features with motor training. During the Recovery phase, two maps were acquired 15 and 30 min after the final training block in order to investigate retention or resolution of any corticomotor adaptations (Figure 1).

The protocol in this study reflected short‐term motor training, in which improvement (“fast learning”) can be induced by a limited number of trials over a time scale of less than one hour (Karni et al., 1998; Luft & Buitrago, 2005). In contrast, long‐term motor training (not assessed) involves slowly evolving incremental performance gains (“slow learning”) triggered by consistent practice over multiple sessions constituting a total timeframe of hours or days (Karni et al., 1998; Luft & Buitrago, 2005).

### 2.3 Lumbopelvic tilt motor training task

Participants sat with their thighs supported, feet flat on the floor, and knees and hips flexed to 90°. Participants allocated to perform the lumbopelvic tilt motor training task had a triaxial accelerometer (CXL10LP3, Crossbow technologies; Weight: 46 g; Dimensions: 1.9 cm × 4.8cm × 2.5cm) affixed to the highest point of the right iliac crest as determined by a trained physiotherapist using palpation. Accelerometer data were sampled at 2 kHz using a Power 1401 data acquisition system and Spike2 software (Cambridge Electronic Design).

Real‐time output of accelerometer motion in the sagittal plane was displayed as a blue line using Spike2 on a computer monitor (53 cm resolution of 1,920 × 1,080) at eye level, two meters in front of the participant. Anterior and posterior tilting of the pelvis was indicated by downward and upward movement of the blue line, respectively. Participants were first instructed to move the blue line to draw a smooth sinusoidal pattern by alternately tilting their pelvis anteriorly and posteriorly as far as possible for 30 s. A red "target" sinusoidal wave was then generated and scaled to 85% of the participant's maximum range in each direction.

Participants allocated to perform the finger abduction (control) task had the accelerometer affixed along the posterior aspect of the right index finger, with the center of the accelerometer positioned over the proximal interphalangeal joint. The forearm was supported in midpronation/supination with the elbow flexed at 90°. Downward and upward movement of the blue trace reflected finger adduction and abduction, respectively. The red target wave was generated as described above. This setup controlled for attention without training lumbopelvic movement or lower back muscle control.

**FIGURE 2:**
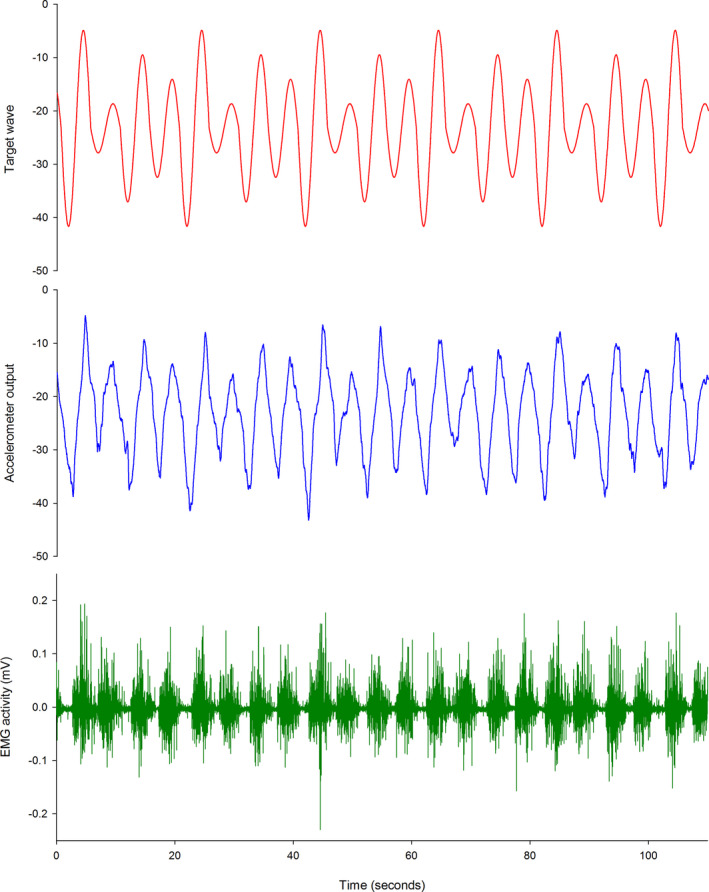
Training data output. Output during training for a representative participant. Red line = target wave, Blue line = participant accelerometer output, Green line = electromyography activity, EMG, electromyography; mV, millivolts

During the recorded trial blocks, the red target wave was presented for 5 min, with the amplitude of the wave altered unpredictably every cycle (25%, 50%, 75%, or 100% of the target range). Participants were instructed to tilt their pelvis (pelvic tilt group) or move their finger (control group) to match the red target wave as accurately as possible. TMS mapping was performed after each 5 min block. A sample of the output during lumbopelvic tilt training for a representative participant is presented in Figure 2.

### 2.4 Electromyography

A pair of bipolar surface electrodes (Ag‐AgCl, Noraxon dual electrodes, interelectrode distance 2.0 cm) was placed over the right LES, 3cm lateral to the spinous process of L3 (Larivière, Arsenault, Gravel, Gagnon, & Loisel, 2003; Schabrun, Jones, Cancino, & Hodges, 2014). The ground electrode was placed over the contralateral anterior superior iliac spine. Electromyographic signals were amplified (×2,000), band‐pass filtered (20–1,000 Hz), and sampled at 2 kHz along with accelerometer data.

### 2.5 Transcranial magnetic stimulation (TMS) mapping

Single‐pulse TMS mapping over the motor cortex was used to investigate corticomotor organization and excitability. Monophasic stimuli were delivered using a Magstim BiStim^2^ (Magstim Co. Ltd). Due to issues with coil availability, two coils were used, either a 70‐mm D70^2^ coil (*n* = 26) or a 70‐mm figure‐of‐eight coil (Magstim Co. Ltd; two age‐ and gender‐matched participants in each group). The coil was placed tangentially to the skull with the handle pointing posteriorly, inducing a current in the posterior–anterior direction (Rossini et al., 2015; Schabrun et al., 2014). As motor‐evoked potentials (MEPs) are difficult to elicit in paraspinal musculature at rest, all stimuli were delivered during submaximal contraction of the LES (O’Connell, Maskill, Cossar, & Nowicky, 2007). To determine submaximal contraction intensity, participants performed three repetitions of a maximal back extension effort against manual resistance. Each contraction lasted 3 s. The target LES EMG during TMS mapping was set at 20% of the highest root mean square (RMS) EMG amplitude for 1 s during the maximal efforts (Tsao, Danneels, & Hodges, 2011). Visual feedback of EMG was provided to ensure that participants maintained the target contraction during the mapping procedure.

The "hotspot" was defined as the coil position that evoked a maximal peak‐to‐peak MEP in the target muscle at a given stimulation intensity (Rossini et al., 2015). The active motor threshold (aMT) was defined as the minimum TMS intensity required to elicit at least five discernible MEPs in a train of ten stimuli delivered to the hotspot during a submaximal LES contraction (Groppa et al., 2012). A Brainsight stereotactic frameless neuronavigation system (Rogue Research Inc) was used to help determine the hotspot and aMT. To maximize participant comfort and allow stimulation to be tailored to each participant's level of corticomotor excitability, the stimulation intensity was set at 120% of the aMT. This contrasts with the maximal stimulator output used in previous studies of lower back muscle representations in M1 and was made possible via use of the high‐intensity 70‐mm D70^2^ coil (Tsao et al., 2011). All procedures are reported in accordance with the TMS‐specific methodological assessment checklist (Chipchase et al., 2012).

During TMS mapping, 90 stimuli were delivered pseudorandomly to the scalp over a 5 × 7 cm grid (6 rows and 8 columns) oriented to the cranial vertex (Cavaleri et al., 2018; van de Ruit et al., 2015). The grid was superimposed on a generic brain image in the neuronavigation display. The cranial vertex was determined using the 10/20 international EEG Electrode Placement system and registered using Brainsight (Herwig, Satrapi, & Schönfeldt‐Lecuona, 2003). The grid extended from 0 to 5 cm laterally and −2 to 5 cm anteriorly (vertex = 0, 0 cm). Additional rows or columns were added if MEPs were recorded at the boundary of the 5 × 7 cm grid during a preliminary assessment in which 30 stimuli were delivered around the perimeter of the grid (Jonker et al., 2019). Stimuli were distributed evenly throughout the grid using a 4‐s interstimulus interval. The neuronavigation display was monitored to ensure that two successive stimuli were not delivered in close proximity and that the entire grid was used.

### 2.6 Data processing

#### 2.6.1 Motor performance data

Motor performance data were processed using MATLAB (MathWorks Inc) based upon the approaches reported by Holland, Murphy, Passmore, and Yielder (2015) and van de Ruit and Grey (2019). Data for the participant's movement and the target wave were extracted every 10 ms, and the mean absolute distance between these traces at each time point was calculated as the motor error. A mean motor error closer to zero reflected greater accuracy during the motor training task.

#### 2.6.2 TMS data

Maps were generated offline using MATLAB. Motor‐evoked potential (MEP) amplitudes were calculated as the RMS EMG amplitude between visually identified onset and offset, from which the background RMS EMG amplitude (55–5 ms preceding stimulation) was subtracted (Tsao et al., 2011). MEP amplitudes were normalized to the peak MEP amplitude during the first baseline map for each participant. Triangular linear interpolation was used to create a full surface map within a transformed plane containing stimulation coordinates and their corresponding MEP amplitudes (Cavaleri et al., 2018; D’Errico, 2005; van de Ruit et al., 2015). The resultant map was divided into 2,500 partitions (50 × 50), with each partition assigned an approximate value based on the nearest acquired MEP. Partitions with MEP amplitudes exceeding 25% of a participant's peak response were labeled as "active." To calculate map area, the number of active partitions was divided by 2,500 and multiplied by the size of the stimulated area (35 cm^2^ for 25 participants, and 40 cm^2^ for 5 participants) (Cavaleri et al., 2018; van de Ruit et al., 2015). Map volume was determined by summing the approximated MEP amplitudes (in millivolts) of all active partitions in the matrix. The center of gravity (CoG), or amplitude weighted center of the map, for each muscle was calculated using the formula: CoG = Σ(*xz*)/Σ*z*; Σ(*yz*)/Σ*z* (where *x* = mediolateral coordinate; *y* = anteroposterior coordinate; and *z* = corresponding MEP amplitude) (Cavaleri et al., 2018; van de Ruit et al., 2015; Wassermann et al., 1992). Shifts in the location of the CoG were calculated as the Euclidean distance (ED) from baseline using the formula: ED = $\sqrt{{\left(y_{1}-y_{2}\right)}^{2}+{\left(x_{1}-x_{2}\right)}^{2}}$ , (where *y* = anteroposterior coordinate; *x* = mediolateral coordinate; and 1 and 2 refer to baseline and post‐training values, respectively) (van de Ruit & Grey, 2019; van de Ruit et al., 2015).

### 2.7 Statistical analyses

Statistical Package for the Social Sciences software (version 23; IBM Corp) was used for all analyses. Statistical significance was set at *p* < .05.

#### 2.7.1 Baseline phase

To examine the within‐session reliability of TMS map features (map volume, area, CoG latitude, CoG longitude) in the absence of motor training, data from the maps acquired during the Baseline phase were compared using absolute intraclass correlation coefficients (ICCs). The ICCs were interpreted with the following values: <0.50 = poor; 0.50–0.64 = moderate; 0.65–0.79 = good; and ≥0.80 = excellent reliability (Cavaleri, Schabrun, & Chipchase, 2017; Higgins & Green, 2015). The second map acquired during this phase was used as the baseline map during the subsequent phases.

#### 2.7.2 Testing and recovery phases

##### Effect of training on motor error and corticomotor reorganization

The effect of lumbopelvic tilt training on motor error was analyzed using a repeated measures analysis of variance (ANOVA) with between‐subject factor "Group" (pelvic tilt versus control) and within‐subject factor "Time" (Block 1, Block 2, Block 3). To examine the effect of lumbopelvic tilt training on corticomotor features (map volume, area, center of gravity location), repeated measures ANOVAs were performed with between‐subject factor "Group" (pelvic tilt versus control) and within‐subject factor "Time" (baseline, post‐training Block 1, post‐training Block 2, post‐training Block 3, 15 min post‐training, and 30 min post‐training).

Assumptions of normality and sphericity for all ANOVAs were assessed using the Shapiro–Wilk test and Mauchly's test of sphericity, respectively (Gamst, Meyers, & Guarino, 2008). The Greenhouse–Geisser correction for nonsphericity was applied for datasets that violated the assumption of sphericity. Where appropriate, post hoc analyses were performed using one‐way repeated measures ANOVAs and Sidak‐adjusted multiple comparison tests.

##### Variability of corticomotor responses to motor training

Baseline map variance was determined by calculating the change in area (Δarea) and volume (Δvolume) between the two maps acquired during the Baseline phase for each individual participant. Participants were classified as positive responders if their change in map area or map volume exceeded the baseline Δarea or Δvolume by greater than 1 *SD*. Conversely, participants were classified as negative responders if their change in map area or map volume was at least 1 *SD* below the baseline Δarea or Δvolume (van de Ruit & Grey, 2019). All other participants were classified as nonresponders. To determine whether responders in the pelvic tilt group had different changes in motor error to nonresponders, repeated measures ANOVAs were conducted with between‐subject factor "Response" (responders versus nonresponders) and within‐subject factor "Time" (Block 1, Block 2, Block 3). Assumptions of normality and sphericity were assessed, and post hoc tests performed, as described previously.

##### Relationship between pelvic tilt task accuracy and corticomotor reorganization

Pearson correlations were used to determine whether changes in task accuracy were correlated with changes in TMS measures. To this end, changes in motor error were regressed against changes in mapping outcomes (map volume, map area, and CoG) recorded after the second and third pelvic tilt training blocks. Change scores were calculated relative to baseline. Pearson correlations were interpreted using the following values: less than 0.30 = poor; 0.30–0.49 = moderate; and greater than or equal to 0.50 = strong association (Cohen, West, & Aiken, 2014).

## 3 RESULTS

### 3.1 Participant characteristics

Participant characteristics are summarized in Table 1. Groups were matched on all baseline variables. Two participants in the pelvic tilt group had an aMT exceeding 84% of maximum stimulator output (MSO) (91% and 93%), and so could not be stimulated at exactly 120% of aMT. These participants were therefore stimulated using 100% MSO. Background EMG activity was well‐maintained between and within maps. The mean RMS EMG preceding stimulation did not differ between groups or over time (Time: *F*_3.9, 110.4_ = 1.4, *p* = .22; Group: *F*_1, 28_ = 1.0, *p* = .33; Group × time: *F*_3.9, 110.4_ = 0.7, *p* = .60). The pulse‐to‐pulse variability of background activity was small (coefficient of variation < 0.25 for all) and was also unaffected by group or time (Time: *F*_6, 168_ = 0.4, *p* = .86; Group: *F*_1, 28_ = 0.2, *p* = .67; Group × time: *F*_6, 168_ = 0.3, *p* = .96). No adverse events were reported.

**TABLE 1:** Participant characteristics

	Pelvic tilt group Mean (*SD*)	Control group Mean (*SD*)	MD (95% CI)	*p*‐value
Sample size (*n*)	15	15	–	
Sex (female, male)	5, 10	5, 10	–	
Right handed (*n*)	13	13	–	–
Age (years)	24.5 (3.1)	25.1 (1.9)	−0.7 (−2.6 to 1.2)	0.48
Height (cm)	173.8 (6.9)	169.3 (6.6)	4.5 (−0.5 to 9.6)	0.08
Weight (kg)	73.2 (13.1)	70.0 (14.3)	3.2 (−7.1 to 13.5)	0.53
Baseline aMT (%)	68.9 (14.9)	60.0 (13.9)	8.9 (−1.9 to 19.7)	0.10

Abbreviations: aMT, active motor threshold; CI, confidence interval; cm, centimeters; kg, kilograms; MD, mean difference; *n*, number; *SD*, standard deviation

### 3.2 Baseline phase

#### 3.2.1 TMS mapping was reliable in the absence of motor training

Map volume (ICC = 0.82 [95% CI 0.66–0.91], *p* < .001) and map area (ICC = 0.87 [95% CI 0.74–0.94], *p* < .001) both demonstrated good‐to‐excellent within‐session reliability prior to the motor training task. These results were consistent with those obtained when maps were not normalized to the peak Baseline MEP amplitude (map volume ICC = 0.87 [95% CI 0.75–0.94]. *p* < .001; map area ICC = 0.84 [95% CI 0.69–0.92], *p* < .001). Center of gravity latitude (ICC = 0.94 [95% CI 0.88–0.97]. *p* < .001) and longitude (ICC = 0.96 [95% CI 0.93–0.98], *p* < .001) demonstrated excellent within‐session reliability.

### 3.3 Testing and recovery phases

#### 3.3.1 Both groups demonstrated improved motor performance

**FIGURE 3:**
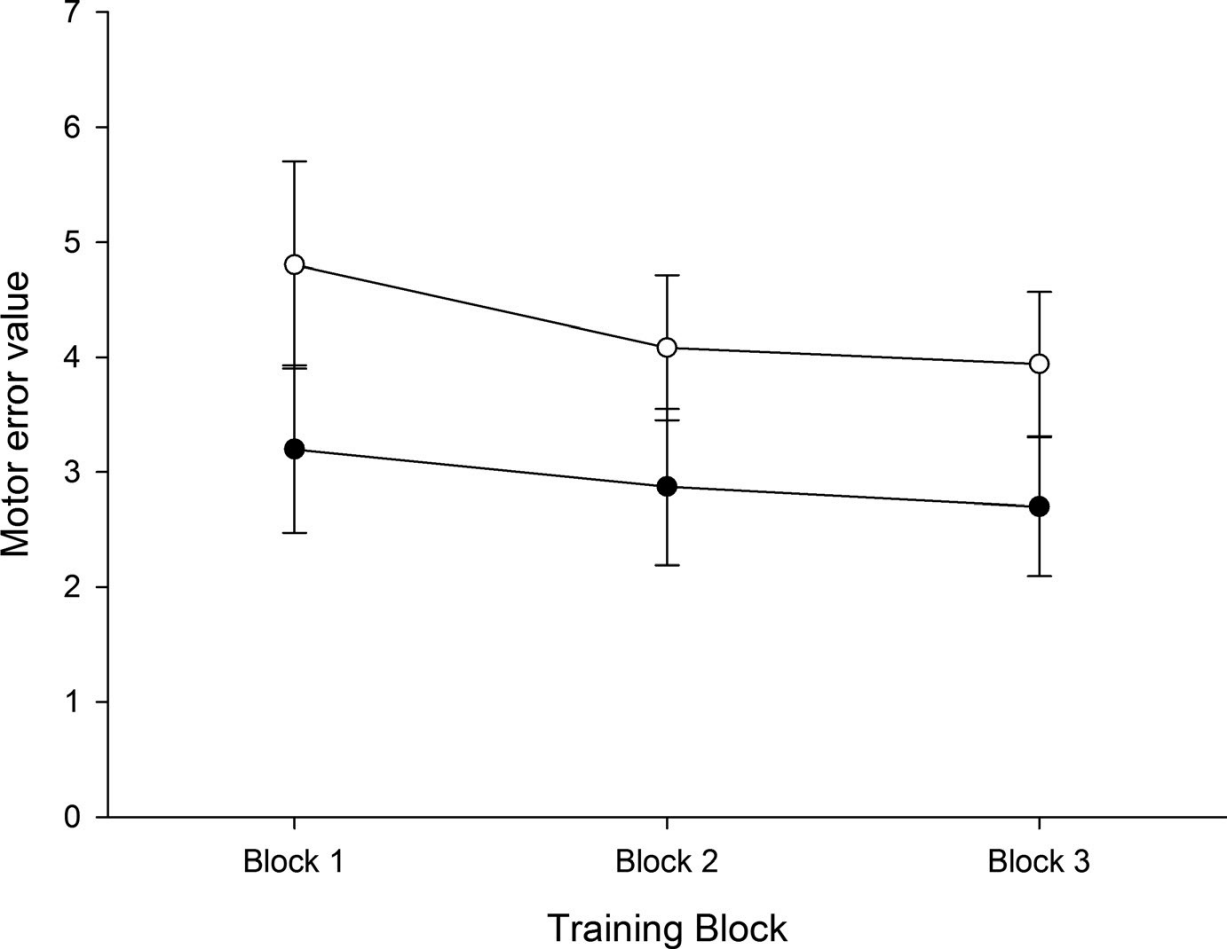
Participant motor error over time. The filled circles represent the pelvic tilt group, and the unfilled circles represent the finger abduction control group. Error bars = standard error

Both the pelvic tilt and control group demonstrated improved performance in the form of reduced motor error over time (Time: *F*_1.3, 35.6_ = 18.5, *p* < .001; Figure 3). Motor error was significantly reduced from Training Block 1 to Training Block 2 (*p* = .002) and from Training Block 2 to Training Block 3 (*p* = .03). Although the control group had a higher degree of motor error at each time point (Group: *F*_1, 28_ = 9.3, *p* = .01, all post hoc *p* = .01), there was no difference between groups in terms of the change in motor error over time (Group × time: *F*_1.3, 35.6_ = 1.7, *p* = .20).

#### 3.3.2 Neither group demonstrated corticomotor reorganization

**FIGURE 4:**
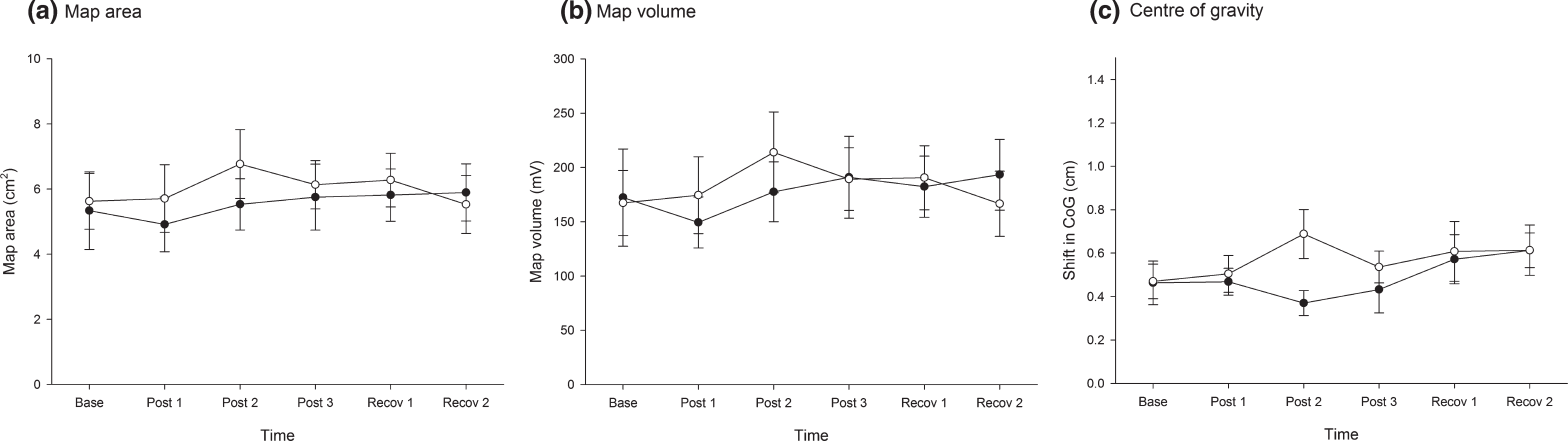
TMS mapping outcomes over time. The filled circles represent the pelvic tilt group, and the unfilled circles represent the finger abduction control group. (a) map area, (b) map volume, (c) center of gravity, Error bars = standard error. Base, baseline; cm, centimeters; mV, millivolts; Post 1, post‐training block 1; Post 2, post‐training Block 2; Post 3, post‐training Block 3; Recov 1, 15 min post‐training; Recov 2, 30 min post‐training

As shown in Figure 4, motor training did not elicit changes in map area (Time: *F*_5, 130_ = 0.7, *p* = .62; Group: *F*_1, 26_ = 0.2, *p* = .65; Group × time: *F*_5, 130_ = 0.5, *p* = .76) or map volume (Time: *F*_5, 130_ = 0.15, *p* = .71; Group: *F*_1, 26_ = 0.05, *p* = .83; Group × time: *F*_5, 130_ = 0.5, *p* = .75) in either group. Similarly, CoG did not change in either group following repeated exposure to the motor training tasks (Time: *F*_5, 130_ = 1.2, *p* = .30; Group: *F*_1, 26_ = 0.6, *p* = .46; Group × time: *F*_5, 130_ = 1.0, *p* = .38; Figure 4).

#### 3.3.3 Corticomotor responses to motor training were variable, and performance did not differ between "responders" and "nonresponders"

The *SD* of the Δarea during the Baseline phase was 2.0 cm^2^, and the *SD* of the Δvolume was 84.2 mV. Participants were therefore categorized as responders in terms of map area if their individual Δarea > 2.0 cm^2^ (positive responder) or <−2.0 cm^2^ (negative responder), and responders in terms of map volume if Δvolume > 84.2 mV (positive responder) or <−84.2 mV (negative responder). All other participants were classified as nonresponders. Although 80% of participants in the pelvic tilt group demonstrated reduced motor error between Training Blocks 1 and 2, only 53% (33% positive, 20% negative) were classified as responders in terms of map area and 53% (40% positive, 13% negative) in terms of map volume over this period. Similarly, 93% of participants in the pelvic tilt group reduced motor error between Blocks 1 and 3, but only 47% (27% positive, 20% negative) were categorized as responders in terms of map area and 53% (33% positive, 20% negative) in terms of map volume. In the control group, 47% (27% positive, 20% negative) of participants were classified as responders for both map area and map volume following Training Block 3. Changes in CoG from baseline were less than 1 cm for 87% of participants in the pelvic tilt group and 93% of participants in the control group.

There was no difference in task performance between participants in the pelvic tilt group classified as responders, and those classified as nonresponders in terms of map area (Time: *F*_2, 26_ = 13.2, *p* < .001; Response: *F*_1, 13_ = 0.5, *p* = .48; Response × Time: *F*_2, 26_ = 0.2, *p* = .84) or map volume (Time: *F*_2, 26_ = 13.7, *p* < .001; Response: *F*_1, 13_ = 0.9, *p* = .37; Response × Time: *F*_2, 26_ = 0.3, *p* = .76).

#### 3.3.4 There was no relationship between changes in pelvic tilt task performance and corticomotor reorganization

**FIGURE 5:**
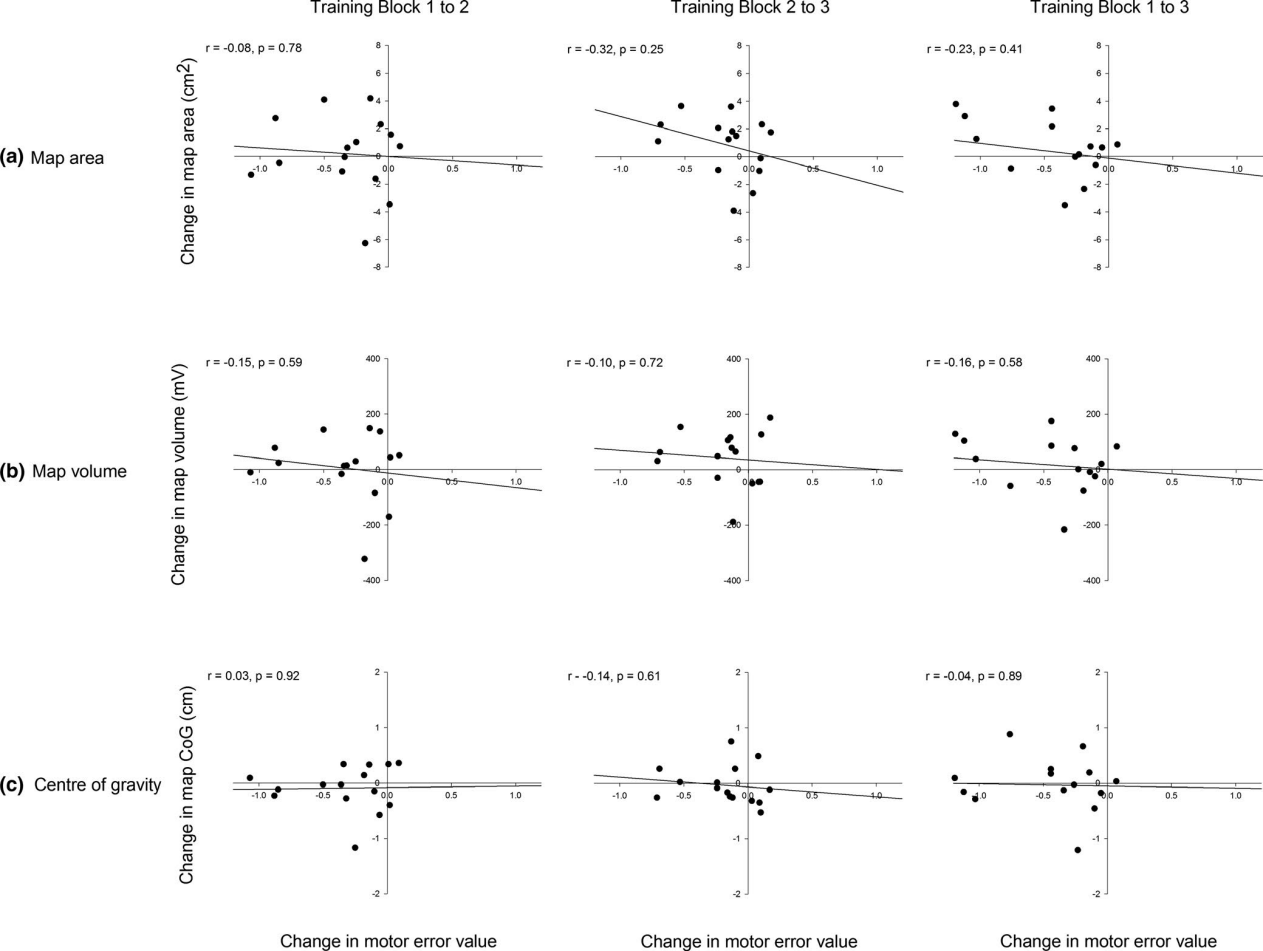
Relationship between task performance and corticomotor reorganization. (a) map area, (b) map volume, (c) center of gravity, cm, centimeters; CoG, center of gravity; mv, millivolts

There was no relationship between changes in pelvic tilt motor error and changes in map area (Block 1–2: *r* = −.08, *p* = .78; Block 2–3: *r* = −.32, *p* = .25; Block 1–3: *r* = −.23, *p* = .41; Figure 5a), map volume (Block 1–2: *r* = −.15, *p* = .59; Block 2–3: *r* = −.10, *p* = .72; Block 1–3: *r* = −.16, *p* = .58; Figure 5b), or CoG displacement (Block 1–2: *r* = .03, *p* = .92; Block 2–3: *r* = −.14, *p* = .61; Block 1–3: CoG: *r* = −.04, *p* = .89; Figure 5c) at any time point.

## 4 DISCUSSION

These data are the first to test for an association between motor performance and reorganization of the corticomotor representations of lower back muscles. Although performance improved during a lumbopelvic tilt visuomotor task, no concomitant changes in map area, volume, or center of gravity were identified by TMS mapping. After three training blocks, only 47% (27% positive, 20% negative) of participants in the pelvic tilt group were classified as responders in terms of map area and 53% of participants (33% positive, 20% negative) in terms of map volume. This suggests that corticomotor adaptations to motor training are variable and may not underpin short‐term motor performance improvements in the lower back.

The protocol used in this study enabled exploration of the effects of short‐term motor training on the corticomotor representations of lower back muscles. A visuomotor tracing task was selected as this type of training has been shown to induce improved motor performance in the hand, upper arm, and ankle joints (Classen et al., 1998; Lotze et al., 2003; Muellbacher et al., 2001; Pascual‐Leone et al., 1994, 1995; Perez, Lungholt, Nyborg, & Nielsen, 2004). Following task familiarization, three training blocks constituting a total of 15 min of motor training were employed and sufficient to reduce motor error. Previous research has demonstrated that as little as 6 min of skill training induces increases in MEP amplitude, with sessions beyond 20 min potentially attenuating corticomotor reorganization due to overlearning or loss of focus (Floyer‐Lea & Matthews, 2004; Jensen, Marstrand, & Nielsen, 2005; van de Ruit & Grey, 2019; Willerslev‐Olsen, Lundbye‐Jensen, Petersen, & Nielsen, 2011). Overlearning describes a process whereby initial increases in MEP amplitude with task performance return to baseline once task mastery has been achieved (Muellbacher et al., 2001). Sequential improvement in performance across three training blocks, but lack of any relationship with TMS map features at any time point, suggests that the findings of this study are not the result of overlearning. Inclusion of the finger abduction group controlled for the attention aspects of the task without training lumbopelvic movement or promoting improved lower back muscle control.

The findings of this study contrast previous reports of corticomotor reorganization following upper limb motor training (Classen et al., 1998; Lotze et al., 2003; Muellbacher et al., 2001; Pascual‐Leone et al., 1994, 1995). A potential explanation for this discrepancy is differences in the neural control underlying hand and lower back movement. Hand muscles are involved in dextrous movements requiring precise control, whereas lower back muscles perform primarily postural functions (Deliagina et al., 2008). Although corticospinal projections to lower back muscles are present in humans (Ferbert, Caramia, Priori, Bertolasi, & Rothwell, 1992), TMS‐evoked responses are small and generally require facilitation via muscle contraction even at high stimulation intensities (Chang et al. 2019; Ferbert et al., 1992; Nathan, Smith, & Deacon, 1996; Tsao, Galea, & Hodges, 2008). Animal studies show greater involvement of the brainstem, vestibulospinal and reticulospinal tracts in control of muscles of the lower back than the hand, which are primarily under cortical control (Deliagina et al., 2008; Galea et al., 2010; Lawrence & Kuypers, 1968; Lemon et al., 2004). The findings of the present study suggest that improved motor performance in the lower back may involve adaptations in these subcortical networks rather than the motor cortex. Treatment techniques promoting corticomotor reorganization may therefore have less impact on motor performance in the lower back than the upper limb. This is supported by recent evidence that peripheral electrical stimulation modifies corticomotor excitability when applied to the upper limb (Barsi, Popovic, Tarkka, Sinkjær, & Grey, 2008; Chipchase, Schabrun, & Hodges, 2011; Golaszewski et al., 2012) but not when applied to the lower back (Elgueta‐Cancino, Massé‐Alarie, Schabrun, & Hodges, 2019).

Another explanation for these findings relates to the nature of the training task itself. The present study used a visuomotor tracing task that required continuous participant attention. In contrast, many studies of upper limb training have used ballistic motor training tasks requiring repeated nonskilled muscle activation (e.g., rapid finger tapping) (Hammond & Vallence, 2006; Ljubisavljevic, 2006; Muellbacher et al., 2001). There is evidence to suggest that different neurophysiological mechanisms may underpin these forms of motor training. For example, inhibitory repetitive transcranial magnetic stimulation over M1 disrupts retention of skill improvements during a ballistic thumb and finger opposition task (Baraduc, Lang, Rothwell, & Wolpert, 2004; Muellbacher et al., 2002), but has no effect on a more complex skill requiring finger movement toward a target while compensating for varying levels of externally applied force (Baraduc et al., 2004). This suggests that M1 may be more engaged during ballistic tasks, potentially having less influence over visuomotor training. During visuomotor training, neural adaptations may occur in regions beyond M1. This is supported by functional neuroimaging studies in both humans and animals demonstrating increases in supplementary motor area, premotor cortex, parietal cortex, and cerebellum activity during complex visuomotor tasks (Catalan, Honda, Weeks, Cohen, & Hallett, 1998; Floyer‐Lea & Matthews, 2004, 2005; Hardwick, Rottschy, Miall, & Eickhoff, 2013).

There is also evidence to suggest that the difficulty of a visuomotor task may influence the degree of corticomotor reorganization that occurs. Previous research suggests that simple tasks can be performed using subcortical circuits alone, eliciting no changes in cortical reorganization (Dhawale, Wolff, Ko, & Ölveczky, 2019; Kawai et al., 2015). Similarly, motor control may be transferred from cortical to subcortical sites once task mastery has been achieved (Hwang et al., 2019). This suggests that the results of the present study could be attributed to a lack of difficulty or complexity in the selected task. However, previous research has shown consistent corticomotor reorganization during similar tracing tasks involving the neck, upper arm, hand, and ankle joints (Lotze et al., 2003; Pascual‐Leone et al., 1994, 1995; Perez et al., 2004; Rittig‐Rasmussen, Kasch, Fuglsang‐Frederiksen, Jensen, & Svensson, 2013). Further, as task mastery was not achieved (93% of participant demonstrated improvements in task performance over time), the results of the present study are unlikely due to a lack of task complexity. Nevertheless, further studies of short‐term motor training in the lower back, involving ballistic motor tasks or visuomotor tasks of varying difficulties, are required to confirm whether the nature of the training task influences the location or extent of cortical reorganization.

Task‐specific corticomotor reorganization may also explain the difference in the results of this study and others involving trunk musculature. For example, repeated voluntary activation of the multifidus or transverse abdominis muscles induces corticomotor reorganization over a two‐to‐three week period (Massé‐Alarie, Beaulieu, Preuss, & Schneider, 2016; Tsao, Galea, & Hodges, 2010). The tracing task used in the present study required coordinated activity of multiple muscles and had greater visuomotor demands than repeated focussed muscle activation, potentially eliciting reorganization in different premotor, subcortical, and spinal centers. Alternatively, given the relatively small influence of corticospinal projections to the lower back, repeated practice over an extended period of time may be required to drive corticomotor adaptations. Indeed, emerging research suggests that task duration may influence the degree and location of cortical reorganization that occurs (Dhawale et al., 2019; Hwang et al., 2019) Further research is required to determine whether reorganization of lower back muscle representations in M1 follows ongoing exposure to complex visuomotor tasks (over periods of days to weeks), or if different schedules of training elicit distinct patterns of corticomotor reorganization. This would provide insight into any potential differences in the neurophysiological mechanisms underpinning short‐term and long‐term motor training. It is also important to note that previous research has been conducted on participants with lower back pain (Massé‐Alarie et al., 2016; Tsao, Galea, et al., 2010), whose corticomotor responses to motor training may differ from those of the healthy participants tested in this study (Schabrun, Burns, Thapa, & Hodges, 2017).

Emerging evidence suggests that the relationship between corticomotor excitability and motor performance may be more variable than once thought. Vallence, Kurylowicz, and Ridding (2013) reported that 28% of participants demonstrated no change in MEP amplitude following a ballistic thumb abduction training task, and more recently, van de Ruit et al. (2019) found that only 18%–36% of participants were classified as responders to a finger visuomotor training task. Consistent with the findings of the present study, these investigations showed no relationship between improved motor performance and corticomotor reorganization. This response variability is also observed following noninvasive brain stimulation paradigms, where "inhibitory" or "excitatory" protocols elicit the predicted corticomotor response in 30%–50% of participants (Fratello et al., 2006; Martin et al., 2006; Müller‐Dahlhaus, Orekhov, Liu, & Ziemann, 2008; Wiethoff, Hamada, & Rothwell, 2014). This variability has been attributed to factors such as differences in anatomy, genetics, and history of synaptic activity (Fratello et al., 2006; Martin et al., 2006; Müller‐Dahlhaus et al., 2008; Ridding & Ziemann, 2010; Wiethoff et al., 2014). The present study extends previous findings by demonstrating no differences in motor performance between individuals who exhibit corticomotor reorganization during training and those who do not. This suggests that, while the neural mechanisms underpinning improved motor performance may vary between participants, this variability does not contribute to observable differences in motor outcomes.

This study employed a rapid TMS mapping technique, rather than averaging a set of MEP amplitudes over a single cranial site, in order to evaluate corticomotor responses to motor training (Cavaleri et al., 2018; van de Ruit et al., 2015). The rapid mapping technique has been previously validated and has shown good‐to‐excellent within‐session reliability when used to map upper limb muscle representations (Cavaleri et al., 2018; van de Ruit et al., 2015; van de Ruit, 2015). The reliability and baseline variance observed in this study are consistent with findings in upper limb representations (van de Ruit & Grey, 2019), and mapping has been shown to produce results consistent with those obtained after averaging MEP responses at the motor cortical hotspot (Summers et al., 2019). Differences between the findings of this study and previous research are therefore unlikely attributable to the mapping technique employed. Despite a rigorous approach toward data collection and analysis, this study is not without limitations. It was not possible to blind the researchers performing TMS mapping to the motor training task as the pelvic tilt and control tasks required different accelerometer placements. This study was also restricted to a small sample of healthy participants. Further research with larger sample sizes, participants with back pain, and a variety of training tasks is required to determine whether the findings reported here have clinical implications.

## 5 CONCLUSIONS

No relationship between corticomotor reorganization and improvements in performance during a lumbopelvic tilt visuomotor task was identified. Substantial variability was observed in terms of corticomotor responses to motor training, with approximately 50% of participants showing no changes in map volume or area despite significant improvements in task performance. These findings suggest that short‐term improvements in lower back motor performance during a visuomotor task may be driven by changes in remote subcortical and/or spinal networks rather than adaptations in corticomotor pathways. However, further research using tasks of varying complexities, schedules, and durations is required to confirm this hypothesis.

## CONFLICT OF INTEREST

None.

## AUTHOR CONTRIBUTIONS

There are no conflicts of interest, additional acknowledgements, or affiliations to report. Rocco Cavaleri, Lucy Chipchase, Hugo Massé‐Alarie, and Paul Hodges were involved in the conception of the study. Rocco Cavaleri, Hugo Massé‐Alarie, Muath Shraim, and Paul Hodges were involved in data collection. All authors were involved in the design, writing, and editing of the study and manuscript. The final manuscript was approved by all authors.

## Data Availability

The data that support the findings of this study are available from the corresponding author upon reasonable request.
